# Transepithelial resistance in human bestrophin-1 stably transfected Madin–Darby canine kidney cells

**DOI:** 10.1080/13102818.2014.988078

**Published:** 2014-12-10

**Authors:** Kirilka Mladenova, Svetla Petrova, Veselina Moskova-Doumanova, Tanya Topouzova-Hristova, Stoyanka Stoitsova, Irena Tabashka, Christina Chakarova, Zdravko Lalchev, Jordan Doumanov

**Affiliations:** ^a^ Faculty of Biology, Sofia University “St. Kliment Ohridski”, Sofia, Bulgaria; ^b^ Institute of Microbiology, Bulgarian Academy of Science, Sofia, Bulgaria; ^c^ Institute of Ophthalmology, UCL, London, UK

**Keywords:** MDCK cells, hBest1, actin, BVMD

## Abstract

Bestrophin-1 (Best1) is a transmembrane protein, found in the basolateral plasma membrane of retinal pigmented epithelial cells. The exact structure and functions of Best1 protein are still unclear. The protein is thought to be a regulator of ion channels, or an ion channel itself: it was shown to be permeable for chloride, thiocyanate, bicarbonate, glutamate and γ-aminobutyric acid (GABA). Mutations in the gene for Best1 are leading to best vitelliform macular dystrophy (BVMD) and are found in several other types of maculopathy. In order to obtain additional information about Best1 protein, we determined cell polarization of a stably transfected Madin–Darby canine kidney cell line II (MDCK II) cell line, expressing human Best1. We measured the transepithelial resistance of transfected and non-transfected MDCK cells by voltmeter EVOM, over 10 days at 24 hour intervals. The first few days (first–fourth day) both cell lines showed the same or similar values ​​of transmembrane resistance. As expected, on the fifth day the non-transfected cells showed maximum value of epithelial resistance, corresponding to the forming of monolayer. The transfected cells showed maximum value of transepithelial resistance on the ninth day of their cultivation. Phalloidin staining of actin demonstrated the difference in actin arrangements between transfected and non-transfected cells due to Best1. As a consequence of actin rearrangement, Best1 strongly affects the transepithelial resistance of polarizing stably transfected MDCK cells. Our results suggest that Best1 protein has an effect on transepithelial resistance and actin rearrangements of polarized stably transfected MDCK cells.

## Introduction

Best vitelliform macular dystrophy (BVMD) is autosomal dominant juvenile onset maculopathy, which is associated with a mutation in human *BEST1* gene.[1,2] *hBEST1* gene encodes bestrophin-1 (Best1) protein, which is expressed basolaterally in retinal pigment epithelium (RPE) [3] and in astrocytes.[4] BVMD involves several stages and leads to loss of central vision. Best1 is a transmembrane protein with molecular weight of ∼68 kDa [5,6] and is thought to be an ion channel: it was shown to be permeable for chloride, thiocyanate, bicarbonate, glutamate and GABA.[4,7–10] The exact structure and functions of the protein and the pathogenesis of BVMD are still under discussion.

Epithelial cells form the boundary surfaces in the body and epithelial cell cultures are used as an *in vitro* model to study the transport of substances through the membrane. Most often in this type of experiments cells are cultivated on a permeable membrane (transwell filters). There they form a continuous layer and cells have apical and basal contact with the cultural medium. Cultivation of cells in these conditions is convenient to study cell polarization, the barrier properties of the layer and the conductivity of the particles (ions).

In a recent study, we used Madin–Darby canine kidney cell line II (MDCK II), stably expressing human Best1 protein and showing the same Best1 localization as in RPE cells.[11,12] These cells are a widely used model for studying of the mechanism of protein sorting and cell polarization,[11,13] since they can be polarized just for about five days.[14] To determine the polarization, non-transfected MDCK and stably transfected Best1 MDCK cells were grown for 10 days on transwells and each day the transepithelial resistance was measured by epithelial voltohmmeter (EVOM) voltmeter. A possible influence of Best1 on the actin cytoskeleton of stably transfected polarized MDCK cells was investigated by fluorescent staining of actin.

## Materials and methods

All reagents and chemicals were supplied by Sigma-Aldrich (Sofia, Bulgaria) unless otherwise stated.

### Cell culture

MDCK II and Best1 stably transfected MDCK [12] cells were grown in Dulbecco's modified eagle's medium (DMEM), in 10% fetal bovine serum, 1% penicillin–streptomycin solution at 310.15 °K and 5% CO_2_. For the transfected cells, 5.10^−4^ kg.l^−1^ G418 as a selective marker was used.

### Measurement of transepithelial resistance

Transepithelial resistance of stably transfected and non-transfected MDCK cells was determined at 24 hour intervals for 10 days. The cells were seeded at an initial concentration of 2.5 × 10^5^ cells/well in six-well transwells filters. The measurements were performed by voltmeter EVOM (World Precision Instruments, Inc), according to manufacturer's instructions.

### Fluorescence staining of actin

Transfected and non-transfected cells were grown on cover slips for seven days with initial concentration of 1 × 10^5^ cells/well. Each day the cells were washed with 1× PBS (phosphate buffer saline) (containing 1 × 10^−4^ mol.L^−1^ CaCl_2_ and 1 × 10^−3^ mol.L^−1^ MgCl_2_). Cells were fixed for 15 minutes with 4% formaldehyde and were permeabilized with 5% Tween 20 in PBS for 10 minutes. Cells were stained for 45 minutes with phalloidin, conjugated with tetramethylrhodamine (TRITC) (Sigma-Aldrich) and were visualized with Nikon TiU confocal laser scanning microscope and the images were acquired and processed using EZC1 software.

## Results and discussion

Since in Best1 stably transfected MDCK cells, Best1 protein does not influence cell growth and cell polarity,[12] we investigated transepithelial resistance of transfected and non-transfected MDCK cells in respect to their polarization for 10 days.

**Figure 1.**
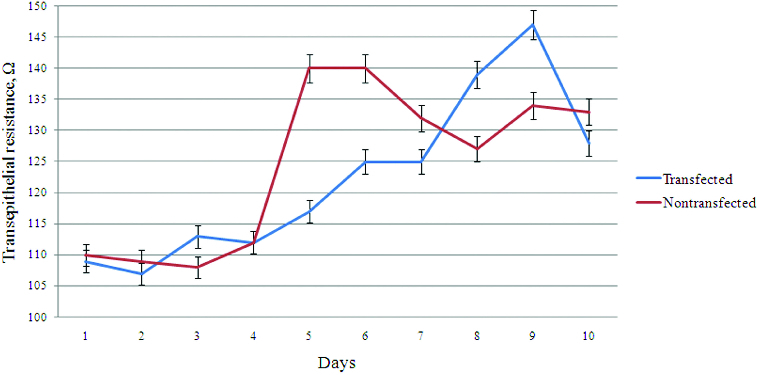
Transepithelial resistance of Best1 transfected and non-transfected MDCK II cell lines. The first few days (first–fourth day) both cell lines showed similar values ​​(about 110 Ω) of transmembrane resistance. On the fifth day, the non-transfected cells showed maximum value ​​(about 140 Ω) of epithelial resistance, corresponding to the formation of monolayer. The transfected cells showed maximum value of transepithelial resistance on the ninth day of their cultivation. Each value represents the mean ± standard error of the mean (SEM) (*n* = 3), and *P* > 0.05 for fourth day and *P* < 0.001 for fifth day.

As is shown in Figure 1, at the beginning of cultivation (first–fourth day) both cell lines showed the same or similar values ​​of transmembrane resistance. As we demonstrated previously by staining for tight junction marker ZO-1,[12] the transfected and non-transfected cells form tight contacts around the fifth–sixth day of cultivation, and therefore, we concluded that these cells were polarized. We can assume that around the fourth day, there are well-formed tight junctions between cells,[12] so the leakage of ions between two cells is disrupted, which could be identified by the lower resistance. From the fourth to seventh day, lower values of transmembrane resistance were detected in stably transfected cells compared to higher values in non-transfected cells. This could be explained by the presence of large amounts of Best1 (as an ion channel) in the transfected cells, and the increased passage of ions in the extracellular space through it. From the seventh to tenth day, stably transfected cells showed a higher transmembrane resistance with values ​​similar to those of the fourth–seventh day in the non-transfected cells. The increase in resistance may be due to the depletion of the intracellular pool of ions (Cl^−^) and equilibration in the ion transport involving Best1. Equilibration in ion transport in non-transfected cells after the formation of tight junction contacts and the establishment of polarity may explain the slight decrease in transepithelial resistance.

Whether the Best1 affect polarization of MDCK cells is important, according to the observation of the more rapid achievement of a higher resistance of the non-transfected cells (for five days) compared with stable transfected (for eight–nine days) cells. This raises the question whether the measured resistance is a result of the polarization of the cells, or is an effect of increased number of Best1 molecules, associated with increased conductivity of ions across the membrane. Increased resistance could not always be determined by increased polarity. It is possible that another mechanism exists that reduces/retards the resistance in stably transfected cells.

**Figure 2.**
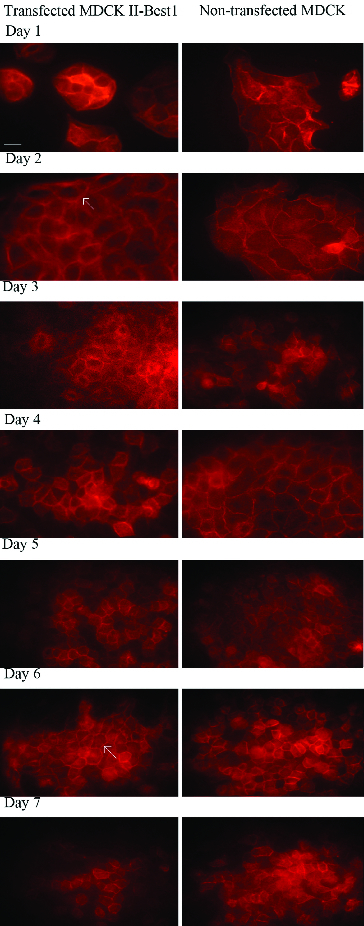
Rearrangement of actin cytoskeleton of Best1 transfected and non-transfected MDCK cells. Transfected and non-transfected cells were grown on cover slips for seven days and actin was stained with phalloidin conjugated with TRITC. From the first to the fifth day, transfected cells showed thicker actin cortex (marked with white arrow on the second day), compared to the non-transfected cells. Actin filaments in both cell lines look morphologically equal on sixth and seventh day. Scale bar – 10 μm.

As an ion channel or regulator of ion channels, Best1 protein could cause reorganization of the actin cytoskeleton and influence conductivity of ions through tight junctions.[15] In order to examine the possible effect of Best1 on actin cytoskeleton reorganization, transfected and non-transfected cells were stained with phalloidin (Figure 2). From the first to the fifth day, transfected cells showed thicker actin cortex compared to non-transfected cells, respectively. On sixth and seventh day, actin filaments in both cell lines look morphologically equal.

This corroborates well with the results of transepithelial resistance (Figure 1) and might suggests the influence of Best1 on actin cytoskeleton rearrangements. As a result, the membranes of the adjacent cells may not form such a ‘tight’ contact, and will be slightly pulled so that the space formed between them would be insufficient for the passage of proteins, but sufficient for the passage of ions.

## Conclusions

Although Best1 protein does not influence cell growth and cell polarity of transfected MDCK cells, these cells showed transepithelial resistance delay which correlates well with the rearrangement of actin cytoskeleton in different days.
